# Innovative vaccine delivery strategies in response to a cholera outbreak in the challenging context of Lake Chilwa. A rapid qualitative assessment

**DOI:** 10.1016/j.vaccine.2017.10.108

**Published:** 2018-10-22

**Authors:** Leonard W. Heyerdahl, Bagrey Ngwira, Rachel Demolis, Gabriel Nyirenda, Maurice Mwesawina, Florentina Rafael, Philippe Cavailler, Jean Bernard Le Gargasson, Martin A. Mengel, Bradford D. Gessner, Elise Guillermet

**Affiliations:** a Agence de Médecine Préventive, Abidjan, Cote d’Ivoire; b University of Malawi, College of Medicine, Community Health Department, Blantyre, Malawi; c Agence de Médecine Préventive, Ferney-Voltaire, France; d Ministry of Health Malawi, Lilongwe, Malawi; e Agence de Médecine Préventive, Paris, France

**Keywords:** Cholera vaccines, Self administration, Fishermen, Vulnerable population, Attitude to health, Anthropology

## Abstract

A reactive campaign using two doses of Shanchol Oral Cholera Vaccine (OCV) was implemented in 2016 in the Lake Chilwa Region (Malawi) targeting fish dependent communities. Three strategies for the second vaccine dose delivery (including delivery by a community leader and self-administration) were used to facilitate vaccine access.

This assessment collected vaccine perceptions and opinions about the OCV campaign of 313 study participants, including: fishermen, fish traders, farmers, community leaders, and one health and one NGO officer. Socio-demographic surveys were conducted, In Depth Interviews and Focus Group Discussions were conducted before and during the campaign.

Some fishermen perceived the traditional delivery strategy as reliable but less practical. Delivery by traditional leaders was acceptable for some participants while others worried about traditional leaders not being trained to deliver vaccines or beneficiaries taking doses on their own. A slight majority of beneficiaries considered the self-administration strategy practical while some beneficiaries worried about storing vials outside of the cold chain or losing vials. During the campaign, a majority of participants preferred receiving oral vaccines instead of injections given ease of intake and lack of pain. OCV was perceived as efficacious and safe. However, a lack of information on how sero-protection may be delayed and the degree of sero-protection led to loss of trust in vaccine potency among some participants who witnessed cholera cases among vaccinated individuals.

OCV campaign implementation requires accompanying communication on protective levels, less than 100% vaccine efficacy, delays in onset of sero-protection, and out of cold chain storage.

## 1 Introduction

Lake Chilwa is located in the southeast region of Malawi, with 32% of its water catchment area in Mozambique. The main activities in and around the lake include fishing, farming, and small-scale business, the Lake Chilwa ecosystem is critical to food security in the region, with an estimated 1.5 million people depending on the lake for their livelihood [1]. In 2015, like other Malawians, Lake Chilwa residents suffered from a “Maize crisis” due to drought and flooding that resulted in maize flour shortages and doubling of selling prices as compared to 2014 [2].

Lake Chilwa has experienced recurrent cholera outbreaks since the 1980s [3], which has a high impact on the fishing communities [4]. The most recent outbreak was reported in December 2015; the index case was a fisherman residing in *zimboweras* (floating homes on the lake). Based on the positive results of a recent OCV campaign organized in Nsanje District, Southern Malawi in March and April 2015 [5], the Ministry of Health (MoH) decided to organize a reactive oral cholera vaccine (OCV) campaign with support from the World Health Organisation (WHO), Médecins Sans Frontières (MSF), and Agence de Médecine Préventive (AMP), collaborating as an interagency OCV group. The OCV campaign was implemented in the Lake Chilwa area between February and March 2016 and targeted fishermen on the lake and the population living within a 2 km radius (lake shore). Innovative strategies were implemented to reach the populations that were at high risk and difficult to access.

From January to March 2016 we organized and conducted a rapid qualitative assessment prior to and during the campaign to investigate anticipated and observed acceptability of OCV and innovative delivery strategies among the fishing-dependent communities targeted by the reactive campaign.

## 2 Methods

### 2.1 OCV campaign and vaccine delivery

The cholera vaccine is usually delivered through two vaccination rounds at least two weeks apart, under medical supervision and requiring a cold chain [6]. The OCV campaign in Malawi used a two-dose delivery strategy. The first round of the campaign was held between 16 and 22 February 2016 and begun the same day in the three settings (shore, islands and *zimboweras*). The second round of the campaign begun on March 8, 2016 on the islands and March 9, 2016 on the shore.

For the first round of the campaign, OCV doses were administrated by health workers as per standard. The second dose was administered using three delivery strategies: (1) For residents living on the shores of the lake, the second dose was administered by health workers as per standard. (2) For those residing in *zimboweras* on the lake, the second dose was given to the individual in a Ziplock bag at the time of the first dose for subsequent unsupervised self-administration. (3) For residents on the islands, community leaders and Health Surveillance Assistants (HSAs) delivered and observed intake of the second dose, but beneficiaries were also able to take doses home for their household members provided they had the appropriate vaccination cards from the first round (a strategy termed “Community-led self-administrated”).

### 2.2 Study site and population

The rapid assessment was carried out before and during the campaign, with four data collection rounds between January and March 2016. The sample was divided between the geographical areas that were to receive the three different OCV delivery strategies, namely Lake Chilwa Shore (Machinga district), *zimboweras* (in Machinga and Zomba districts), and Chisi and Chinguma island (Zomba district).

**Fig. 1:**
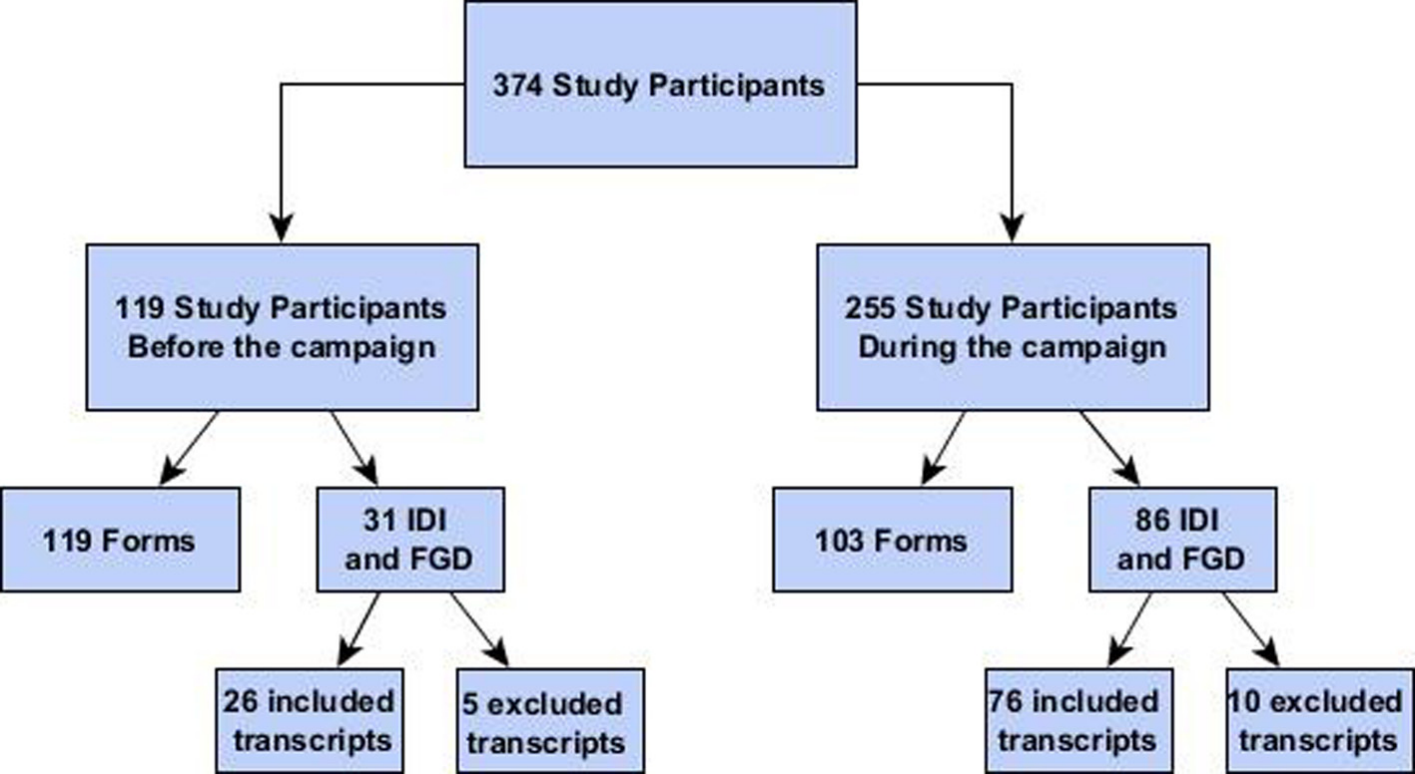
Study participants flowchart.

The sampling strategy was purposive, based on the respondent’s experience with cholera and the profiles and roles sought in the assessment. Participants were recruited using the snowball technique [7]. In total, 313 participants were included (see Fig. 1 and Table 1).

**Table 1:** Number of transcripts and participants who participated to the anthropological assessments conducted before and during the OCV campaign implemented in February and March 2016 in Lake Chilwa area, Malawi.

Sources type	Transcripts	Participants
Number of In Depth Interviews before the campaign	15	15
Number of Focus Group Discussions before the campaign	11	77

**Total transcripts and participants before the campaign**	**26**	**92**
Number of In Depth Interviews during the campaign	52	52
Focus Group Discussions during the campaign	24	169

**Total transcripts and participants during the campaign**	**76**	**221**
**Total transcripts and participants**	**102**	**313**

### 2.3 Characteristics of respondents

Laypersons included fishermen, fishermen’s wives, fish traders, and farmers. Community leaders included village chiefs, religious chiefs, and heads of village committees. Community health agents included volunteers and HSAs. At the regional response level, a local resident working for an international organization was included (see Table 2).

**Table 2:** Characteristics of participants attending In Depth Interviews (IDI) and Focus Group Discussions (FGD), anthropological assessment, March 2016, Lake Chilwa Area, Malawi.

	Pre-campaigns assessments						Assessments during campaign					
	Shore		Islands		Zimboweras		Shore		Islands		Zimboweras	
	KII	FGD	KII	FGD	KII	FGD	KII	FGD	KII	FGD	KII	FGD
Fishermen	1	14	2	21	1	21	7	27	8	44	6	22
Female head of a fishing household	3	14	1	7	0	0	4	14	3	43	0	0
Fish transformers and traders	0	0	0	0	2	0	0	7	1	0	1	0
Farmer not presently fishing	1	0	0	0	0	0	2	12	0	0	0	0
Community leaders	1	0	1	0	0	0	2	0	13	0	0	0
Community health workers	2	0	0	0	0	0	1	0	3	0	0	0
Response officer	0	0	0	0	0	0	1	0	0	0	0	0

Total	8	28	4	28	3	21	17	60	28	87	7	22

### 2.4 Data collection tools and analysis

A short questionnaire, aimed at collecting social characteristics and key elements regarding perceptions of cholera and vaccines, was administered via in-depth interviews (IDI) and focus group discussions (FGD) with participants. IDIs based on a semi-structured guide were carried out with 67 participants. All IDIs were conducted with participants who gave written, informed consent. All interviewees were individuals who were judged to have relevant knowledge about cholera in the community or vaccination programmes in Malawi.

Overall, 35 FGDs were held with community members (laypersons) in the targeted area. FGDs were facilitated by two trained research assistants. One led the discussion, while the other took notes. The FGDs were tape-recorded, fully transcribed, translated, and imported into Nvivo©. All sources were coded using predetermined categories, leaving the possibility for code creation to allow for unexpected, emergent themes.

Data entry and analyses were performed using Epi Info 7. Descriptive analyses were conducted to provide frequency distributions and test the associations as necessary.

### 2.5 Ethical approval

The study protocol was approved by the Malawi National Health Sciences Research Committee (reference number NHSRC # 15/5/1599). All interviewees provided written, informed consent after reading or having been read the participant information sheet.

## 3 Results

### 3.1 Living condition

According to laypersons, communities around Lake Chilwa engage mostly in subsistence farming and small-scale fishing on foot and by dugout canoe.

Lake Chilwa is officially open for fishing from March 1 to December 1, although some fishing is still carried out during the period when the lake is officially closed. A few participants have suggested that the food shortages and high prices meant that the population were not getting enough to eat, so the lake was more crowded in late 2015 and early 2016 than in previous years.

Lake Chilwa includes several islands with an estimated but fluctuating population of 14,000. The lake also includes floating homes, the *zimboweras*. Used as temporary shelters, made of phragmites (tall grass), they are built by fishermen on the shallow parts of the lake. Most residents are men, with a few female traders and sex workers. Fishermen on the islands or on the shore tend to live with their families; some, but not all, use the *zimboweras*. Movement occurs between the family residence, the *zimboweras*, the islands, and the markets, with residency on the lake ranging from one day to three months, rarely even more. Some fishermen operating on Lake Chilwa also operate on other lakes at other times of the year, but this is rare and most mobility is limited to Lake Chilwa.

On the *zimboweras,* fishermen communities have no access to safe water and sanitation or electricity (except for limited solar panels). There are no formal health structures such as village health committees on the lake. Visits from health personnel or NGOs, dependent on boats and large amounts of gasoline, reportedly only occurred during outbreaks. Clusters of *zimboweras* usually include a so-called “tea room”, which is larger than the other *zimboweras* and acts as a central point where daily goods are sold and food can be bought and cooked. The tea room owner is described as influential. During the OCV intervention, tea rooms were used as a delivery point for vaccines and Water, Sanitation and Hygiene (WASH) items, and the owners acted as local focal points.

### 3.2 Social context of cholera before the implementation of the OCV campaign

The *Zimboweras* were identified as the starting point of the 2015 outbreak by members of the lake area communities and the health authorities, which elicited calls for the fishermen’s eviction. According to community leaders living on the shore and the islands, half of the fishermen in transit on the floating homes agreed to leave and not return for the duration of the outbreak; the other half decided to stay and defend their floating homes. This conflict, spurred by the outbreak, culminated with a cluster of *zimboweras* being burned by angry villagers in Namanja. As a result, before the campaign, some fishermen stated being afraid when they saw motorboats circling their *zimboweras* and people on board taking pictures (without communicating why) in early January 2016. Some fishermen reported that rumors were spreading about potential forced eviction. One rumor in particular suggested that the lake had been sold to Mozambique and that a vaccine campaign (by injection) would be organized to hurt the fishermen. Aside from these rumors, the participants were eager to receive OCV (see Table 3).

**Table 3:** Oral Cholera Vaccine (OCV) anticipated acceptability January 2016, Lake Chilwa Area, Malawi.

(n 119)	Yes	No	NA^*^
	N (*%)*	N (*%*)	N (*%*)
*Would you accept to be vaccinated against cholera?*			
On the shore	49/49	0/49	0/49
On islands	36/37	1/37	0/37
In zimboweras (floating homes)	32/33	1/33	0/33
Overall	117/119	2/119	0/119

*Do you have children?*			
On the shore	42/49	7/49	0/49
On islands	33/27	3/37	1/37
In zimboweras (floating homes)	25/33	8/33	0/33
Overall	106/119	12/119	1/119

*If you have children would you want them to be vaccinated?*			
On the shore	42/48	0/48	6/48
On islands	32/33	0/33	1/33
In zimboweras (floating homes)	20/25	0/25	5/25
Overall	94/106	0/106	12/106

### 3.3 OCV acceptability

Before the campaign, OCV acceptability was very high, with 100% of respondents on the shore (n = 49), 97% on the islands (n = 36), and 97% in the *zimboweras* (n = 32; Annexes, Table 3) declaring they would accept the vaccine if it were introduced. In the interviews, a couple of participants explained that others may potentially refuse the vaccine fearing that it may reduce fertility. During the campaign, the vast majority of participants received, or were planning or willing to receive the vaccine doses. Only a few refused the vaccine, due to vomiting after the first dose; the feeling that old age would make them immune to cholera; and for another participant, the fear that the vaccine was a contraceptive, “not sexually safe”. For all three second dose strategies, several participants indicated that vaccination dates, and where applicable sites, were changed or not communicated leading to missed doses of OCV.

All participants declared that receiving the vaccine would not negatively impact their own water and sanitation practices. However, half of them declared that other community members may behave less hygienically as a consequence. For example, one participant indicated:

*“When I arrived here this week, I asked a woman for water to drink since I was thirsty. I asked why she gave me untreated water and she said that ‘we received the cholera vaccine last week so no need to treat the water since we’re protected’.”*

Female fish trader, Chinguma (FGD CD_0703_01)

### 3.4 Perception on vaccine delivery strategies

**First strategy: Directly Observed Vaccination (DOV), two doses delivered by HSAs** (used on the shore). This was perceived as a good strategy by the majority of participants. However, one fisherman from the floating homes indicated that having two distinct days of vaccine delivery may be problematic, because he would need to come back to the shore twice.

*“The best strategy is the one where they give you the second dose that you administer yourself. For example in my case, I would have been thinking of going back to Kachulu (shore) if I could have taken the second dose myself.”*

Female fish trader, zimbowera (IDI_ SM_1203_01)

**Second strategy: Community-Led Self-Administrated Second Dose** (used on the islands). This was considered a good strategy by some participants on the islands, one on the lake shore and a few in the *zimboweras*. However, a few participants from the islands noted that the use of Ziplock bags may be a “bad idea”, as they thought that all vaccine users should take OCV under direct medical supervision. One participant mentioned that relying on traditional leaders was only possible if they were trained:

*“If the community leaders are well- trained then they can be given a chance to help us take the vaccine and taking two doses of vaccine at different times”*

Woman, Chisi island (IDI_AZ_0903_04)

Another participant said that changing previously scheduled dates for the second vaccination round worried him.

**Third strategy: Self-Administration Strategy** (deployed on the *zimboweras*). Interviewees on the islands predominantly perceived this strategy as problematic. Among interviewees on the shore, about half thought this a feasible strategy provided participants received correct instructions on how to store and take the vaccine. Perceived risks included potential vaccine loss, vaccine storage outside the cold chain, and non-professionals opening vials 14 days after the first round. Almost half of participants from the *zimboweras* would have preferred a more traditional strategy expressing concern about vaccine storage:

*“The best way was to take the first dose and then choose a person here [zimboweras] to keep the second dose. It isn’t safe for everyone to keep the vaccine by themselves; depending on the places where they live, some of these places may become very hot, maybe sun is coming inside, so it is not safe.”*

Fisherman, zimbowera (IDI_ LH_1003_02)

### 3.5 Perception of vaccine administration technique

Perceptions of safety and efficacy drove participant preference for vaccine administration technique. A third of participants before the campaign and a ninth during the campaign stated that they had no preference for the mode of administration; for most of them only efficacy mattered. While a third of participants reported a preference for each of oral and injectable vaccine delivery before the campaign, after the campaign preference for oral delivery increased to two-thirds and that for injectable decreased to a quarter with more participants mentioning pain at the injection site:

*“Some adults also hate being pierced with needles because of the pain so oral is best.”*

Wife of a fisherman, Namanja (FGD_JM_1103_01)

Participants noted that vaccines delivered via injection had an advantage because “they go straight into the blood”. One participant noted that vaccine delivered by injection would be long lasting:

“Injectable will pass on in blood vessels and stay there for a long time compared to oral which is effective for a short time.”

Fisherman, zimbowera (FGD_AP_3001_01)

In contrast to those who preferred injectable vaccines, some participants declared that oral vaccine protection lasts longer than injected vaccines.

A few interviewees noted that injections may lead to injuries by accident, incompetency, or the deliberate action of vaccine providers, for example:

*“There were rumors that the lake has been sold…by injecting people, it would be something to eliminate them so that the fishermen would go (leave the lake). So the oral vaccine is more acceptable than the injectable (…) The injected one, they say maybe is like you would be sucking their blood and maybe poisoning them.”*

Fisherman, Zimbowera (IDI_LH_3001_01)

A few participants stated that oral vaccine may be easier or faster to administer, notably if it could be self-administered. Another side effect of injectable vaccines cited was a reduction in fertility:

*“Some would not come if you were using injections because they believe that using a syringe would make their sex ineffective.”*

Fisherman, Zimbowera (FGD_SM_1602_00)

Some participants, predominantly before the campaign, explained that oral vaccines were for children, while injectable vaccines were for adults. Likewise, a few participants noted that vaccines for toddlers did not match those for adults in terms of their nature or the “power” of the vaccine dose. During the campaign, several participants said that the oral vaccine was “easy to administer to kids” or less painful for them.

Several HSAs reported that some Chichewa speakers may have misunderstood the nature of OCV because of international stakeholders’ use of the word “cholera” in printed materials. “Cholera” reads as “tcholera” in Chichewa and can literally be understood as “it is a contraceptive” (“cho” meaning it is, “lera” an abbreviation of “kulera”)). The word *Kulera*, which means to “rock a baby to sleep” has been used to describe family planning in Chichewa. The Chichewa word for cholera (the disease) is spelt *Kolera*.

### 3.6 Perception of vaccine efficacy

A majority of participants thought the vaccine had or will have efficacy. However, half the participants before the campaign and a quarter during, expressed uncertainty regarding the efficacy of OCV saying that they “did not trust the vaccine” because “people get cholera even after receiving the first dose”.

### 3.7 Perceptions of vaccine safety

Before the campaign, most participants noted that they were ready to receive the vaccine, that they trusted the government or the scientists, or that they would have to wait and see if there were potential side effects. A couple of participants noted that they had to follow the government’s decision regarding the use of OCV. During the campaign, the majority of participants stated they “did not feel any side effects”. Only a few participants declared that they had personally experienced minor side effects after taking an OCV dose, including dizziness, diarrhea, stomach pain, nausea, or vomiting. Several others mentioned that they heard of or saw others in the community experiencing these minor side effects. A couple of participants stated that the vaccine “worsens the situation” or “weakens the body and lowers immunity” when someone becomes sick with cholera. As noted above, one participant refused to take the vaccine until being guaranteed it was not compromising fertility:

*“All my children and my wife during pregnancy receive all vaccines, but not this cholera one, unless you tell me its importance and assure me it is sexually safe. I have not received any of the OCV doses.”*

Fisherman, Chisi island (FGD_JN_0903_01)

Most respondents indicated that OCV has a “new taste” or a “bad taste” like “rotten eggs” or “burnt chicken”, is “salty” or “sour”, or “tasted like bad milk”. Despite having experienced the bad taste, almost all interviewed participants (with the exception of two) indicated that the bad taste would not deter them from taking future doses of OCV, because: (1) it is a medicine, which is not supposed to taste good; and (2) it protects against cholera.

## 4 Discussion

This first ever qualitative study on self- and community-led self-administration of OCV found high pre- and post-campaign acceptability of both strategies in Malawi’s Lake Chilwa area . The anticipated 98% acceptance of OCV aligns with previous research on anticipated [9], [10] and observed [5] acceptance conducted in other settings. Preferred vaccine delivery strategy seemed predicated on: (1) the possibility of preserving the integrity of OCV and (2) independence and ease of access by self-administering the second dose. Overall, participants perceived the strategies from which they benefitted to be better than the other strategies in the targeted area. While designed for mobile fishermen, mobility posed a problem for self-administration due to possible loss or overheating of the vaccine if kept in the user’s pocket or exposed to direct sunlight in a canoe during fishing.

The results concur with previous research that vaccine coverage in underserved regions could be increased by the use of non-needle and increasingly thermostable vaccines [11] and self-administration [12]. The findings also confirm the need to identify and include hard-to-reach and vulnerable groups, such as fishermen, to ensure that OCV is delivered equitably [9], [13]. Results stress the need for logistical and sociological assessments [8] to identify locally pertinent stakeholders (such as tea room owners), bottlenecks to acceptability (pre-campaign rumors, reactions to cases during the campaign) and, practical hurdles to vaccine preservation (mobility of fishermen in practice).

Finally, results emphasize the need to take findings from such assessments and incorporate them into campaign designs and pre-campaign communication tools. Additionally, public health officials should be aware of available information that can assist with campaign design. For example, recent studies assessing OCV safety and efficacy when vaccines are stored outside the cold chain [14] may be used as a basis for communication to community representatives and the population when a self-administration strategy is implemented. Similarly, studies showing efficacy of orally administered vaccines, including OCV, could be used in social messaging.

This would have addressed one of the important findings from our study, namely that lack of communication on lack of 100% vaccine efficacy and the delay between administration and protection likely contributed to some loss in vaccine confidence among participants witnessing cholera cases among vaccinated individuals. Likewise, the misunderstandings on the words cholera, *kolera* and *kulera* suggest the need for ongoing vigilance in all communications during the campaign and about unexpected barriers to vaccine uptake.

On a more general basis, we recommend that the international community and public health officials support implementation science and take advantage of results when designing and implementing immunization strategies including campaigns [15]. This will be particularly important when using new strategies (such as self-administered vaccines), improving immunization coverage in areas with historically low levels, deciding between substantially different vaccines (such as injectable versus oral vaccines), and working with targeted beneficiary groups with logistical challenges (such as mobile and/or hard to reach groups) or socio-cultural conditions that might influence vaccine acceptance.

### 4.1 Study limitations

We used a purposive sampling technique to select IDIs and FGDs participants, so our findings might not be fully representative of the targeted population. While our study sought to determine the range of common issues associated with cholera and OCV, our study was not comprehensive enough to determine the full range and importance of different issues. As a descriptive study, we could not determine factors associated with specific responses, which makes the design of targeted interventions more problematic.

## 5 Conclusions

OCV was highly accepted among residents of Lake Chilwa. Acceptability, uptake, and continued WASH practices could be increased through communication on the level of protection afforded by the vaccine, the delay in seroprotection, and appropriate vaccine storage options for the self-administration strategy. Mass vaccination campaigns, especially those using new vaccines, can benefit from prior studies to increase the understanding and reach of the target population.
