# Supplementary material for: Innovative vaccine delivery strategies in response to a cholera outbreak in the challenging context of Lake Chilwa. A rapid qualitative assessment
Source: Vaccine. 2018 Oct 22;36(44):6491–6. doi: 10.1016/j.vaccine.2017.10.108 (PMC6189868; doi:10.1016/j.vaccine.2017.10.108)
Supplement: Supplementary data 1 [file mmc1.docx]

**Data collection tools for the qualitative assessment of Lake Chilwa OCV campaign, January-March 2016**

Agence de Médecine Préventive (AMP)

Polytechnic College of Blantyre

COLLABORATORS:

- Ministry of Health (MOH), Malawi
- University of Malawi Polytechnic (UMP)
- Agence de Médecine Préventive (AMP)

PRINCIPAL INVESTIGATORS:

- AMP: Mr. Leonard Heyerdahl
- UMP: Dr Bagrey Ngwira

CO-PRINCIPAL INVESTIGATOR:

- AMP: Dr Elise Guillermet

CO- INVESTIGATORS:

- UMP: Mr. Gabriel Nyirenda
- AMP: Ms Rachel Demolis
- MOH: Mr Maurice M. Mbang’ombe

Nota bene: The data collection tools were developed in the emergency context of a cholera outbreak in Lake Chilwa area. The assessment accompanied a reactive Oral Cholera Vaccine campaign conducted by the Ministry of Health using three different delivery strategies in February-March 2016.

Contents

[1. Interview Canvas in communities on the shore of the lake (strategy 1) 2](#_Toc489992274)

[2. Interview Canvas in communities in floating homes (strategy2) 6](#_Toc489992275)

[3. Interview Canvas in communities in islands (strategy 3) 10](#_Toc489992276)

[4. Interview Canvas for Local health providers 13](#_Toc489992277)

[5. Interview Canvas for health response agents regional, national or international level 17](#_Toc489992278)

[6. Short Questionnaire 18](#_Toc489992279)

### 1. Interview Canvas in communities on the shore of the lake (strategy 1)

| **Investigator = _ _ Interviewee ID : __ __ __ Date dd/mm: __ __ / _ _ / 16** |
| --- |

1. **General information for all interviewees :**

- **(Gender)-> Questionnaire**
- **Age (years) -> Questionnaire**
- **Marital Status**
- **Country of Origin**
- **Main activity: Fishing - Conditioning fish - fish sales - Agriculture - Office - business - Service (mechanic, electrician), student, housewife, not active ,retired**
- **Revenues: which regular and temporary expenses? Which kind of revenues used? Revenues from whom (in household/ outside household such as migrants or NGO…)?**

1. **Political organization and community engagement**

- **In your communities (families) who makes the final decisions?**
- **Which committees are available in your community? What is their role?(probe for specific roles for each committee)**
- **Which stakeholders are working in your community? What is their role?**

1. **Vaccines and immunization activities**

- **Perceptions of vaccine**
  - **Do you think vaccines are Safe (probe for direct experience of AEFI?)**
  - **In your perception do you think vaccines are effective? (Efficacy)**
  - **How do you feel that vaccines should have a specific target (Depending on target)**
  - **How do feel about diseases new vaccines against old vaccines (Depending on preventable diseases)**
  - **Do you prefer oral vaccine or as injectable (probe the reason for each)[Depending on delivery techniques (i.e. injectable/oral) (to be explained)]**
- **Practices and reasons for**
  - **Do you make ensure that your Children are fully-vaccinated or not and why?**
  - **Do you ensure that Pregnant women are fully-vaccinated or not and why (i.e. knowledge, perceptions or barriers for vaccine access)**
  - **Do you ensure that children/women are vaccinated against all preventable diseases**
  - **Which vaccines do you usually receive (injectables/oral)**
- **In your opinion, which is the best delivery strategies for vaccine**
  - **Routine versus campaign**
  - **Fixed versus mobile (preferable site to be cited)**
  - **Health workers versus volunteers**
  - **Time for campaign (i.e. period during the year, day during the week, moment during the day)**
  - **Island staying and vaccine campaigns, what solution in place and time ?**

1. **Cholera**

- **(If it doesn’t appear specifically before) Have you ever heard about cholera? -> Questionnaire**
- **If so, could you describe this disease?**
- **If so, do you think Cholera is dangerous?**
- **Have you or someone you know ever had cholera?**
- **Could you describe what happened to her/him (treatments, healing/death, events following the onset of symptoms, how long they stay in bed or they were not able to work …)?**
- **Did you receive information on cholera prevention/protection from officials or NGO?**
  - Probe ( information in the health center, health officer came to the village, radio information, newspaper, NGO, poster)
- **Did you participate in a study for cholera previously (questions, simple)**
  - **If yes, when and with who**
- **If yes, according to officials or NGO, what do you have to do to protect yourself? And what do you think about the usefulness of these messages? And feasibility of these propositions?**
  - **About washing hands before meals**
  - **About washing hands after going to the toilets**
  - **About the main source of drinkable water : surface well, deep well, rain water, bottled water, piped-water (direct access or paid to another person)**
- **How do you think you can get cholera? -> Questionnaire**

Biomedical (Bacteria in unclean water), punishment for a fault or sorcery. Ask if there can be several causes for cholera and which they are, how to know which cause.

- **How do you protect yourself from cholera? -> Questionnaire**
  - **Difference floating homes / Island/ along the shore**
- **How do you usually treat cholera and why?**
  - **Efficiency, accessibility and cost considerations**

**(Probe if there are any difference in floating homes / Island/along the shore**

- **What do you think is the best way to treat cholera?**

1. **What about oral cholera vaccine?**

- **Have you ever heard about Oral cholera vaccine?**
  - **IF YES Who informed you of the vaccine (probe radio, friend, church, health worker)**
    - **What did they say?**
- **Have you taken a first dose of the vaccine?**
  - **IF YES, where did you take it? Was it far from where you were?**
    - **What date did you take it?**
    - **Can you describe how you took or were given the first dose?**
    - **Who gave you the first dose? do you think it was the right person to deliver vaccine ?**
    - **How did the vaccine taste like? Does the taste compromise you taking another does in the future?**
    - **Did you feel side effects after the first dose? if yes, describe**
  - **IF NOT, why (probe not aware of campaign, was too far, unadvised by friends, don’t need it, prefer someone else has it)**

| **IF THE INTERVIEW IS CONDUCTED BEFORE THE SECOND DOSE TIME PERIOD (before March 5)**   - **Will you take the second dose?**   - **IF YES, do you know how it will be delivered?**     - **Do you know when this second dose will be available?**     - **Who will provide you with the dose?**     - **What do you think of this delivery organization? (probe rather have medically assisted or having the second dose given during the first round or given by community leader).**   **IF NOT, why (probe side effects first dose, taste, unadvised by friends, don’t need it, does not have a dose or know where to get it, prefer someone else has it)** |
| --- |
|  |

| **IF THE INTERVIEW IS CONDUCTED DURING OR AFTER THE SECOND DOSE TIME PERIOD (>March 5)**   - **Did you take the second dose ?**   - **IF YES,**      - **What date did you take it?**     - **Can you describe how you took or were given the second dose?**     - **Who gave you the second dose? do you think it was the right person to deliver vaccine ?**     - **What was the difference between the first and second dose ?**     - **Which delivery mode did you prefer ? explain**     - **How did the vaccine taste like? does the taste compromise you taking another does in the future?**     - **What do you think of this delivery organization? (probe rather have medically assisted or having the second dose given during the first round or given by community leader).**   **IF NOT, why (probe side effects first dose, taste, unadvised by friends, don’t need it, does not have a dose or know where to get prefer someone else has it, delivery mode not adapted to me)** |
| --- |

**FOR ALL INTERVIEWEES**

- **what expectation do you have as regards the efficacy of the vaccine (ie. notably for 1 dose versus 2 doses)**
- **How long will the protection last**
- **If you took or will take the vaccine do you think it changes something as regards to handwashing or what kind of water you get?**
- **Do you think other people in the community might change their way of handling water after vaccination? explain.**
- **Expectations for vaccine safety and AEFI monitoring**
  - **Sometimes vaccines can present adverse effects to certain individuals, who do you think should be involved in AEFI monitoring?**
  - **Did you receive specific instructions to report these possible adverse events**
    - **If yes, explain who informed you and what they recommended**
    - **Where (i.e. in health facility, at home?)**
- **Expectations for vaccine target**
  - **Who you expect the cholera vaccine should target?**
    - **Children versus adult**
    - **The mos**t vulnerable population versus the entire population

### 2. Interview Canvas in communities in floating homes (strategy2)

| **Investigator = _ _ Interviewee ID : __ __ __ Date dd/mm: __ __ / _ _ / 16** |
| --- |

1. **General information for all interviewees :**

- **(Gender)-> Questionnaire**
- **Age (years) -> Questionnaire**
- **Marital Status**
- **Country of Origin**
- **Main activity: Fishing - Conditioning fish - fish sales - Agriculture - Office - business - Service (mechanic, electrician), student, housewife, not active ,retired**
- **Revenues: which regular and temporary expenses? Which kind of revenues used? Revenues from whom (in household/ outside household such as migrants or NGO…)?**

1. **Political organization and community engagement**

- **In your communities (families) who makes the final decisions?**
- **Which committees are available in your community? What is their role?(probe for specific roles for each committee)**
- **Which stakeholders are working in your community? What is their role?**

1. **Vaccines and immunization activities**

- **Perceptions of vaccine**
  - **Do you think vaccines are Safe (probe for direct experience of AEFI?)**
  - **In your perception do you think vaccines are effective? (Efficacy)**
  - **How do you feel that vaccines should have a specific target (Depending on target)**
  - **How do feel about diseases new vaccines against old vaccines (Depending on preventable diseases)**
  - **Do you prefer oral vaccine or as injectable (probe the reason for each)[Depending on delivery techniques (i.e. injectable/oral) (to be explained)]**
- **Practices and reasons for**
  - **Do you make ensure that your Children are fully-vaccinated or not and why?**
  - **Do you ensure that Pregnant women are fully-vaccinated or not and why (i.e. knowledge, perceptions or barriers for vaccine access)**
  - **Do you ensure that children/women are vaccinated against all preventable diseases**
  - **Which vaccines do you usually receive (injectables/oral)**
- **In your opinion, which is the best delivery strategies for vaccine**
  - **Routine versus campaign**
  - **Fixed versus mobile (preferable site to be cited)**
  - **Health workers versus volunteers**
  - **Time for campaign (i.e. period during the year, day during the week, moment during the day)**
  - **Island staying and vaccine campaigns, what solution in place and time ?**

1. **Cholera**

- **(If it doesn’t appear specifically before) Have you ever heard about cholera? -> Questionnaire**
- **If so, could you describe this disease?**
- **If so, do you think Cholera is dangerous?**
- **Have you or someone you know ever had cholera?**
- **Could you describe what happened to her/him (treatments, healing/death, events following the onset of symptoms, how long they stay in bed or they were not able to work …)?**
- **Did you receive information on cholera prevention/protection from officials or NGO?**
  - Probe ( information in the health center, health officer came to the village, radio information, newspaper, NGO, poster)
- **Did you participate in a study for cholera previously (questions, simple)**
  - **If yes, when and with who**
- **If yes, according to officials or NGO, what do you have to do to protect yourself? And what do you think about the usefulness of these messages? And feasibility of these propositions?**
  - **About washing hands before meals**
  - **About washing hands after going to the toilets**
  - **About the main source of drinkable water : surface well, deep well, rain water, bottled water, piped-water (direct access or paid to another person)**
- **How do you think you can get cholera? -> Questionnaire**

Biomedical (Bacteria in unclean water), punishment for a fault or sorcery. Ask if there can be several causes for cholera and which they are, how to know which cause.

- **How do you protect yourself from cholera? -> Questionnaire**
  - **Difference floating homes / Island/ along the shore**
- **How do you usually treat cholera and why?**
  - **Efficiency, accessibility and cost considerations**

**(Probe if there are any difference in floating homes / Island/along the shore**

- **What do you think is the best way to treat cholera?**

1. **What about oral cholera vaccine?**

- **Have you ever heard about Oral cholera vaccine?**
  - **IF YES Who informed you of the vaccine (probe radio, friend, church, health worker)**
    - **What did they say?**
- **Have you taken a first dose of the vaccine?**
  - **IF YES, where did you take it ? Was it far from where you were?**
    - **What date did you take it?**
    - **Can you describe how you took or were given the first dose?**
    - **Who gave you the first dose? do you think it was the right person to deliver vaccine ?**
    - **How did the vaccine taste like? Does the taste compromise you taking another does in the future?**
    - **Did you feel side effects after the first dose? if yes, describe**
  - **IF NOT, why (probe not aware of campaign, was too far, unadvised by friends, don’t need it, prefer someone else has it)**
- **Did you receive a second dose?**
  - **If Yes describe**
    - **What instructions were you given (to open it, when to take it, to store it)**
    - **Where do you keep it? is it complicated for you to keep a dose ?**
  - **If not received 2^nd^ dose but interviewee received first dose**
    - **Why did you not receive the second dose (out of stock, took first dose elsewhere)**

| **IF THE INTERVIEW IS CONDUCTED BEFORE THE SECOND DOSE TIME PERIOD (before March 5)**   - **Will you take the second dose?**   - **IF NOT, why (probe side effects first dose, taste, unadvised by friends, don’t need it, does not have a dose or know where to get it, prefer someone else has it)** - **What do you think of this delivery organization? (probe rather have medically assisted or having the second dose given during the first round).** |
| --- |
|  |

| **IF THE INTERVIEW IS CONDUCTED DURING OR AFTER THE SECOND DOSE TIME PERIOD (>March 5)**   - **Did you take the second dose ?**   - **IF YES,**      - **What date did you take it?**     - **Can you describe how you took or were given the second dose?**     - **Who gave you the second dose? do you think it was the right person to deliver vaccine ?**     - **What was the difference between the first and second dose ?**     - **Which delivery mode did you prefer ? explain**     - **How did the vaccine taste like? does the taste compromise you taking another does in the future?**     - **What do you think of this delivery organization? (probe rather have medically assisted or having the second dose given during the first round).**   **IF NOT, why (probe side effects first dose, taste, unadvised by friends, don’t need it, does not have a dose or know where to get prefer someone else has it, delivery mode not adapted to me)** |
| --- |

**FOR ALL INTERVIEWEES**

- **what expectation do you have as regards the efficacy of the vaccine (ie. notably for 1 dose versus 2 doses)**
- **How long will the protection last**
- **If you took or will take the vaccine do you think it changes something as regards to handwashing or what kind of water you get?**
- **Do you think other people in the community might change their way of handling water after vaccination? explain.**
- **Expectations for vaccine safety and AEFI monitoring**
  - **Sometimes vaccines can present adverse effects to certain individuals, who do you think should be involved in AEFI monitoring?**
  - **Did you receive specific instructions to report these possible adverse events**
    - **If yes, explain who informed you and what they recommended**
    - **Where (i.e. in health facility, at home?)**
- **Expectations for vaccine target**
  - **Who you expect the cholera vaccine should target?**
    - **Children versus adult**
    - **The mos**t vulnerable population versus the entire population

### 3. Interview Canvas in communities in islands (strategy 3)

| **Investigator = _ _ Interviewee ID : __ __ __ Date dd/mm: __ __ / _ _ / 16** |
| --- |

1. **General information for all interviewees :**

- **(Gender)-> Questionnaire**
- **Age (years) -> Questionnaire**
- **Marital Status**
- **Country of Origin**
- **Main activity: Fishing - Conditioning fish - fish sales - Agriculture - Office - business - Service (mechanic, electrician), student, housewife, not active ,retired**
- **Revenues: which regular and temporary expenses? Which kind of revenues used? Revenues from whom (in household/ outside household such as migrants or NGO…)?**

1. **Vaccines and immunization activities**

- **Perceptions of vaccine**
  - **Do you think vaccines are Safe (probe for direct experience of AEFI?)**
  - **In your perception do you think vaccines are effective? (Efficacy)**
  - **How do you feel that vaccines should have a specific target (Depending on target)**
  - **How do feel about diseases new vaccines against old vaccines (Depending on preventable diseases)**
  - **Do you prefer oral vaccine or as injectable (probe the reason for each)[Depending on delivery techniques (i.e. injectable/oral) (to be explained)]**
- **Practices and reasons for**
  - **Do you make ensure that your Children are fully-vaccinated or not and why?**
  - **Do you ensure that Pregnant women are fully-vaccinated or not and why (i.e. knowledge, perceptions or barriers for vaccine access)**
  - **Do you ensure that children/women are vaccinated against all preventable diseases**
  - **Which vaccines do you usually receive (injectables/oral)**
- **In your opinion, which is the best delivery strategies for vaccine**
  - **Routine versus campaign**
  - **Fixed versus mobile (preferable site to be cited)**
  - **Health workers versus volunteers**
  - **Time for campaign (i.e. period during the year, day during the week, moment during the day)**
  - **Island staying and vaccine campaigns, what solution in place and time ?**

1. **Cholera**

- **(If it doesn’t appear specifically before) Have you ever heard about cholera? -> Questionnaire**
- **If so, could you describe this disease?**
- **If so, do you think Cholera is dangerous?**
- **Have you or someone you know ever had cholera?**
- **Could you describe what happened to her/him (treatments, healing/death, events following the onset of symptoms, how long they stay in bed or they were not able to work …)?**
- **Did you receive information on cholera prevention/protection from officials or NGO?**
  - Probe ( information in the health center, health officer came to the village, radio information, newspaper, NGO, poster)
- **Did you participate in a study for cholera previously (questions, simple)**
  - **If yes, when and with who**
- **If yes, according to officials or NGO, what do you have to do to protect yourself? And what do you think about the usefulness of these messages? And feasibility of these propositions?**
  - **About washing hands before meals**
  - **About washing hands after going to the toilets**
  - **About the main source of drinkable water : surface well, deep well, rain water, bottled water, piped-water (direct access or paid to another person)**
- **How do you think you can get cholera? -> Questionnaire**

Biomedical (Bacteria in unclean water), punishment for a fault or sorcery. Ask if there can be several causes for cholera and which they are, how to know which cause.

- **How do you protect yourself from cholera? -> Questionnaire**
  - **Difference floating homes / Island/ along the shore**
- **How do you usually treat cholera and why?**
  - **Efficiency, accessibility and cost considerations**

**(Probe if there are any difference in floating homes / Island/along the shore**

- **What do you think is the best way to treat cholera?**

1. **What about oral cholera vaccine?**

- **Have you ever heard about Oral cholera vaccine?**
  - **IF YES Who informed you of the vaccine (probe radio, friend, church, health worker)**
    - **What did they say?**
- **Have you taken a first dose of the vaccine?**
  - **IF YES, where did you take it ? Was it far from where you were?**
    - **What date did you take it?**
    - **Can you describe how you took or were given the first dose?**
    - **Who gave you the first dose? do you think it was the right person to deliver vaccine ?**
    - **How did the vaccine taste like? Does the taste compromise you taking another does in the future?**
    - **Did you feel side effects after the first dose? if yes, describe**
  - **IF NOT, why (probe not aware of campaign, was too far, unadvised by friends, don’t need it, prefer someone else has it)**

| **IF THE INTERVIEW IS CONDUCTED BEFORE THE SECOND DOSE**   - **Will you take the second dose?**   - **IF YES, do you know how it will be delivered?**     - **Do you know when this second dose will be available?**     - **Who will provide you with the dose?**     - **What do you think of this delivery organization? (probe rather have medically assisted or having the second dose given during the first round).**   **IF NOT, why (probe side effects first dose, taste, unadvised by friends, don’t need it, prefer someone else has it)** |
| --- |

| **IF THE INTERVIEW IS CONDUCTED DURING OR AFTER THE SECOND DOSE**   - **Did you take the second dose ?**   - **IF YES,**      - **What date did you take it?**     - **Can you describe how you took or were given the second dose?**     - **Who gave you the second dose? do you think it was the right person to deliver vaccine ?**     - **What was the difference between the first and second dose ?**     - **Which delivery mode did you prefer ? explain**     - **How did the vaccine taste like? does the taste compromise you taking another does in the future?**     - **What do you think of this delivery organization? (probe rather have medically assisted or having the second dose given during the first round).**   **IF NOT, why (probe side effects first dose, taste, unadvised by friends, don’t need it, prefer someone else has it, delivery mode not adapted to me)** |
| --- |

**FOR ALL INTERVIEWEES**

- **what expectation do you have as regards the efficacy of the vaccine (ie. notably for 1 dose versus 2 doses)**
- **How long will the protection last**
- **If you took or will take the vaccine do you think it changes something as regards to handwashing or what kind of water you get?**
- **Do you think other people in the community might change their way of handling water after vaccination? explain.**
- **Expectations for vaccine safety and AEFI monitoring**
  - **Sometimes vaccines can present adverse effects to certain individuals, who do you think should be involved in AEFI monitoring?**
  - **Did you receive specific instructions to report these possible adverse events**
    - **If yes, explain who informed you and what they recommended**
    - **Where (i.e. in health facility, at home?)**
- **Expectations for vaccine target**
  - **Who you expect the cholera vaccine should target?**
    - **Children versus adult**
    - **The mos**t vulnerable population versus the entire population

### 4. Interview Canvas for Local health providers

| 1. **General information for all interviewees :**  - **General information for all interviewees :** - **(Gender)-> Questionnaire** - **Age (years) -> Questionnaire** - **Marital Status** - **Country of Origin** - **Main activity in health** - **Name of the associated Health Center**   - **whom (in household/ outside household such as migrants or NGO…)?** P - **P**lace in the health system -Head of work force- Leader of professional group- Official injection provider- Unofficial injection provider- Health worker who does not provide injections - Not a health worker, but an informal injection provider- Other- Public -private - Seniority  1. **Political organization and community engagement in the community in which he/she operates.**  - **Social history, organization and rifts** - **Health promotion/community engagement organization**   - **Who are credible leaders and why (i.e. social status, based on what kind of social links/rules?)**   - **Process of involvement (i.e. who decides and why?)**   - **Compensation (i.e. type and amount? who provides the budget?)**   - **Role and activities**   - **Incidence on health practices**  1. **Vaccines and immunization activities**  - **Perceptions of vaccine**    - **How do you feel that vaccines should have a specific target (Depending on target)**   - **How do feel about diseases new vaccines against old vaccines (Depending on preventable diseases)** - Perceptions of techniques for immunization   - What is your perception of syringes versus oral vaccine according to the following elements: safety – efficacity? - According to your experiences of immunization activities, could you tell me about the different attitudes of families towards vaccines provided during routine immunization - What are the families' perceptions of immunization activities and their motives for vaccine refusal or acceptance? - What are the Influence of technique ( needle versus oral or other and routine versus campaigns) - Does the content of vaccine appear important? What criteria are used to evaluate the vaccines’ efficiency and safety? - **FOR Oral Cholera Vaccine (Shanchol) in particular** - According to you, what are the advantages and drawbacks of OCV? - *How does it compare in regards to* - Vaccine administration technique and ease of use? - vaccine preparation and anxiety - safety issues such as AEFI and waste management - What are your expectations regarding the OCV efficiency when it comes to   - - - wastage and lost opportunities for vaccination       - logistic issues, including storage and transport       - Impact of untrained health workers recruitment - What constraints do you think might make families avoid health services or not attend vaccination sessions? - Do you encounter *o*rganizational, distribution of infrastructures constraints? - Do you encounter constraints in terms of - Distance and transport- Decision-making process at home -Cost - Do you encounter Health teams’ organization and social factors such as – language barriers- communication difficulties between staff and families - **In your opinion, which is the best delivery strategies for vaccine**   - **Routine versus campaign**   - **Fixed versus mobile (preferable site to be cited)**   - **Health workers versus volunteers**   - **Time for campaign (i.e. period during the year, day during the week, moment during the day)**   - **As a professional who knows vaccines and the communities, what do you think of:**     - - **Classic Two rounds strategy vs Self Administration. Problems and strenghts**       - **Classic Two rounds strategy vs administration via community leaders. Problems and strenghts**       - **Self Administration VS administration via community leader. Problems and strenghts**  1. **Logistics and monitoring**  - General logistics - Where do you keep your stock? - How much storage do you have? - What type of vaccine do you carry? - How large is your stockpile? - Which health center do you depend on for receiving the need vaccine? - How often do you get stocks? - How large is your refrigerated room? - OCV And Logistics - Transportation - How do you make sure there is a minimal amount of breakages is limited during the transportation of the vials? - Do you have contingency plans, ie, do you plan for additional vials in case of breakages? - How do you usually insure to maintain the cold chain during transportation? - Did you *have cases of frozen vials?* - *Quality monitoring* - Do you find the vial monitor easy to read and trustworthy? - Thermostable *Vaccines* - The Shanchol requires to be stocked up to 30 month between 2 and 8 degrees and can be left at room temperature for 14 days: how do you think this can impact your organization? - Would it require extra storage space? - Would it be easier for transportation, especially the last mile? - self- administration and logistic - What do you think giving the vaccines to the population for them to self administrate later on? - Would it alleviate the logistic burden of your center? - What difficulties would you envision? - Waste management - Where do think the vaccine should be discarded? - MSF plans on leaving bins where vials can be discarded. What is your opinion on this strategy? - What is the easiest way to plan the recovery of those empty vials? - Surveillance - What is the current surveillance techniques used in your facility?  What other monitoring techniques have you used in the past?  How important is monitoring the vaccines intakes in the case of Cholera? - In the case of self- administration strategy for the second round, what do you think of the following possibilities? - Leaving bins where on each floating house cluster and collect the discarded vials on day 14 and counting the empty vials? - filling vaccination cards - Filling registers (a dose taken at the health facility is considered taken)  1. **Past experience of working with community representative**  - **Have you previously worked with Community** representative in dealing with immunization? - For which health program? - What is a positive experience? - What impacted your experience? their training- availability- attitudes- trustworthiness?  1. **Past personal experiences of introduction of new health devices**  - Have you already experienced introduction of new health devices? - Was your experience positive? What impacted your experience – provider- Health system organization -Safety of device Efficiency of device-other  1. **Cholera**  - **Have you ever taken charge of a cholera patient?**   - **If yes, What did you do?** - **Is cholera a major problem for the community?** - **In your opinion what are the causes the community cites for cholera?** - **Do you think there is a proper knowledge of cholera in the communities**   - **If not, explain the gaps**   What do you think is the best way to treat cholera?   1. **Possibilities of adopting OCV in the future**  - Could you benefit from real-life experiences and lessons learned from the past introduction of new devices or injection methods, particularly relating OCV, in comparison injectable vaccines? - Are you open to change and willing to implement new practices  1. What would be the determining factors regarding acceptability of the OCV? |
| --- |

### 5. Interview Canvas for health response agents regional, national or international level

| **Investigator = _ _ Interviewee ID : __ __ __ Date dd/mm: __ __ / _ _ / 16** |
| --- |

1. **General information for all interviewees :**

- **(Gender)-> Questionnaire**
- **Age (years) -> Questionnaire**
- **Marital Status**
- **Country of Origin**
- **Main activity in health**

1. **Your organization**

- **What is the role of your organization?**
  - **In general**
  - **In Cholera**
- **What is your personal role and position in the organization?**
- **How long have you been working in Malawi**
- **Have you been working on cholera for a long time?**

**3. OCV Campaign**

- **What do you think of the use of OCV as a tool for cholera control?**
  - **In general**
  - **In this context**
- **Do you think the OCV campaign could have a negative effect on wash practices? explain.**
  - **IF YES, how could these effects be curved in your opinion ?**
- **In your opinion, what were or are the main roadblocks to using the vaccine as a control tool?**
- **Probe (acceptability, process of request ICG, delivery, etc)**
- **In your opinion what was the rationale for deploying innovative strategies in lake Chilwa, versus a classic medically assisted 2 rounds?**
- **Do you think self-administration (strategy 2) is adapted to the context of the floating homes?**
- **Do you think delivery via community leaders (strategy 3) is adapted to the context of islands?**
- **Do you think these innovative strategies could equal or surpass a classic campaign in terms of coverage and acceptability? explain**
- **Do you think the experience of OCV use in Malawi in this campaign could be used for other settings, if so, how so?**

### 6. Short Questionnaire

| **Interviewer = _ _ Interviewee ID : __ __ __ Date dd/mm: __ __ / _ _ / 16** | | |
| --- | --- | --- |
|  | **A ) District : __ __ __ __ B) Job : __ __ __ ____ __ __ C) Tel number __ __ __ __ __ __ __ __** | |
|  | **D) Year of birth : __ __ __ __ E) Gender** *1= female 2= Male* | |
| *1.* | ***Health in the neighborhood*** |  |
| 1.1 | **What diseases affect the neighboorhood?***1=Malaria 2=Diabetes 3=Choléra 4=Polio 5= communautaire 6=Autre, préciser, ……………* |  |
| 1.2 | **Do you know Cholera ?** *0=No 1=Yes* |  |
| 1.3 | **What are Cholera symptoms (what does it do) ?** *1=Fever 2 = diarrhea with blood 3=Diarrhea without blood 4= vomiting 5= Other, specify…………* |  |
| 1.4 | **How can one get Cholera ?** *… 1=Mosquito 2 = dirtiness 3=wind 4=traditional burying 5= dirty water 6= dirty food 7= other, specify………* |  |
| *2.* | ***Whereabouts*** |  |
| 2.1 | **What is the distance to the nearest Health facility ?** *enter KM _ _ _ _ _ __TIME _ _ _ _ _ Name facility _ _ _ _ _ _ _ _* |  |
| 2.2 | **What is the distance to the nearest Market ?** *enter KM _ _ _ _ _ TIME_____Name Market _ _ _ _ _ _ _ _ _ _* |  |
| 2.3 | **What are the days of the Market ?** *1=Monday 2=Tuesday 3= Wednesday 4= Thursday 5= Friday 6= Saturday 7= Sunday* |  |
| *3.* | ***Vaccination*** |  |
| 3.1 | **Would you accept to be vaccinated against cholera ?** *0 =Yes 1 = No* |  |
| 3.1.2 | **If no, list the reasons you do not want this vaccine ?** *0=No need 1=does not work 2=Family member or friend advised against it 3=I prefer injections 4=bad experience with vaccine 5=other* |  |
| 3.2 | **Do you have children?** *0 =Yes 1 = No* |  |
| 3.2.1 | **(If yes), would you accept to receive the anti-cholera vaccine for your children?** *1=Yes 2=No* |  |
| 3.2.2 | **(If no), list the reasons you do not want this vaccine for your children?** *0=No need 1=does not work 2=Family member or friend advised against it 3=I prefer injections 4= not suitable for children 4=fear of side effects 5=I do not trust vaccine makers 6=other* |  |
| 3.3 | ***(If yes to 3.2 or 3.1*), In General, what would be the best days in the week to organize a vaccination?** *1=Monday 2=Tuesday 3= Wednesday 4= Thursday 5= Friday 6= Saturday 7= Sunday* |  |
|  |  |  |
